# *Sinorhizobium meliloti* RNase III: Catalytic Features and Impact on Symbiosis

**DOI:** 10.3389/fgene.2018.00350

**Published:** 2018-08-28

**Authors:** Margarida Saramago, Marta Robledo, Rute G. Matos, José I. Jiménez-Zurdo, Cecília M. Arraiano

**Affiliations:** ^1^Instituto de Tecnología Química e Biológica António Xavier, Universidade Nova de Lisboa, Oeiras, Portugal; ^2^Grupo de Ecología Genética de la Rizosfera, Estación Experimental del Zaidín, Consejo Superior de Investigaciones Científicas, Granada, Spain

**Keywords:** RNA degradation, endoribonucleases, ribonuclease III, *Sinorhizobium meliloti*, symbiosis

## Abstract

Members of the ribonuclease (RNase) III family of enzymes are metal-dependent double-strand specific endoribonucleases. They are ubiquitously found and eukaryotic RNase III-like enzymes include Dicer and Drosha, involved in RNA processing and RNA interference. In this work, we have addressed the primary characterization of RNase III from the symbiotic nitrogen-fixing α-proteobacterium *Sinorhizobium meliloti*. The *S. meliloti rnc* gene does encode an RNase III-like protein (*Sm*RNase III), with recognizable catalytic and double-stranded RNA (dsRNA)-binding domains that clusters in a branch with its α–proteobacterial counterparts. Purified *Sm*RNase III dimerizes, is active at neutral to alkaline pH and behaves as a strict metal cofactor-dependent double-strand endoribonuclease, with catalytic features distinguishable from those of the prototypical member of the family, the *Escherichia coli* ortholog (*Ec*RNase III). *Sm*RNase III prefers Mn^2+^ rather than Mg^2+^ as metal cofactor, cleaves the generic structured R1.1 substrate at a site atypical for RNase III cleavage, and requires higher cofactor concentrations and longer dsRNA substrates than *Ec*RNase III for optimal activity. Furthermore, the ultraconserved E125 amino acid was shown to play a major role in the metal-dependent catalysis of *Sm*RNase III. *Sm*RNase III degrades endogenous RNA substrates of diverse biogenesis with different efficiency, and is involved in the maturation of the 23S rRNA. *Sm*RNase III loss-of-function neither compromises viability nor alters morphology of *S. meliloti* cells, but influences growth, nodulation kinetics, the onset of nitrogen fixation and the overall symbiotic efficiency of this bacterium on the roots of its legume host, alfalfa, which ultimately affects plant growth. Our results support an impact of *Sm*RNase III on nodulation and symbiotic nitrogen fixation in plants.

## Introduction

Ribonucleases (RNases) promote processing and degradation of RNA transcripts to rapidly adjust gene expression according to cellular needs. In the model bacteria *Escherichia coli* and *Bacillus subtilis* much is understood about the catalytic features and the role of RNases in RNA turnover (Arraiano et al., 2010). However, in phylogenetically diverse non-classical model microorganisms the sets of RNases are poorly characterized. The α–proteobacterium *Sinorhizobium meliloti* belongs to the group of agronomically relevant microorganisms generically referred to as rhizobia, which are well-known for their ability to establish species-specific nitrogen-fixing endosymbioses with legume plants (Fisher and Long, 1992). Only a few studies concerning rhizobial RNases have been reported (Madhugiri and Evguenieva-Hackenberg, 2009; Baumgardt et al., 2014, 2016, 2017; Robledo et al., 2015; Saramago et al., 2017). RNase E is regarded as the major single-strand specific endoribonuclease in gram-negative bacteria (Silva et al., 2011). In *S. meliloti*, this RNase was shown to be required for small RNA (sRNA)-mediated post-transcriptional silencing of quorum-sensing and cell cycle related mRNAs (Baumgardt et al., 2014, 2016; Robledo et al., 2015). On the other hand, in gram-positive bacteria, RNA degradation initiates with RNase Y and RNase J1/2 endoribonucleases (Laalami et al., 2014; Durand et al., 2015). Despite the fact that *S. meliloti* is a gram-negative bacterium, it encodes an RNase J-type enzyme with high similarity to *B. subtilis* RNase J2. *S. meliloti* RNase J was suggested to be responsible for the final maturation of the 5′-termini of the two 23S rRNA fragments and of 16S rRNA (Madhugiri and Evguenieva-Hackenberg, 2009). Few years ago, the ultraconserved *E. coli* YbeY protein was characterized as a novel single-strand specific endoribonuclease, whose activity affects ribosome quality control and processing of 16S rRNA 3′-terminus (Jacob et al., 2013). Interestingly, it was recently shown that the *S. meliloti* YbeY ortholog exhibits an unprecedented catalytic versatility, being able to cleave both single- and double-stranded RNA substrates (ssRNA and dsRNA). Further, *S. meliloti* YbeY was shown to promote degradation of mRNAs from transporter genes upon their antisense interaction with the AbcR2 sRNA (Saramago et al., 2017).

RNase III-like enzymes constitute another group of widely conserved endoribonucleases that have emerged as important players in the post-transcriptional control of gene expression in prokaryotes and eukaryotes. This group of enzymes is also able to cleave RNA molecules internally, within double-stranded segments. In eukaryotes, RNase III-like proteins are involved in RNA interference (RNAi) and RNA maturation (Hammond, 2005). In bacteria, RNase III regulates its own synthesis, is responsible for rRNA operon maturation, processing of cellular and phage RNAs, control of plasmid copy number and maturation of type II CRISPR RNAs (Arraiano et al., 2010; Saramago et al., 2014a, 2015). As a double-strand specific endoribonuclease, this enzyme is a major effector of mRNA processing and decay driven by antisense transcription, and participates in regulation by *trans*-acting sRNAs (Lasa et al., 2011; Viegas et al., 2011). Intriguingly, RNase III has also been shown to interact in a protective mode with certain dsRNA species (Blaszczyk et al., 2004; Akey and Berger, 2005; Ji, 2006). Activity of this endoribonuclease influences virulence traits of some pathogenic bacteria. Specifically in *Salmonella*, it is involved in motility, proliferation inside host cells, biofilm development, and antibiotic susceptibility (Viegas et al., 2011, 2013; Saramago et al., 2014b; Matos et al., 2017). Considering the crucial role that RNase III-like proteins play in different organisms, it would be interesting to investigate the function of RNase III in rhizobia. Here, we report on the cofactor-dependent catalytic features of *S. meliloti* RNase III (*Sm*RNase III) and its impact on the overall symbiotic performance of this rhizobial species on alfalfa (*Medicago sativa* L.) roots. Our results provide novel insights into RNase III-dependent catalysis and unravel a major role of this enzyme throughout the symbiotic interaction.

## Materials and Methods

### Sequence Alignment and Phylogenetic Tree Construction

RNase III sequences from *S. meliloti* (Uniprot ID: M4MQR5), *Rhizobium* sp. (Uniprot ID: C3M8S4), *Agrobacterium tumefaciens* (Uniprot ID: Q8UGK2), *Rhodobacter capsulatus* (Uniprot ID: Q52698), *Pseudomonas aeruginosa* (Uniprot ID: Q9XCX9), *E. coli* (Uniprot ID: P0A7Y0), *Salmonella enterica* (Uniprot ID: Q56056), *Staphylococcus aureus* (Uniprot ID: Q2FZ50), *B. subtilis* (Uniprot ID: P51833), *Streptococcus pneumoniae* (Uniprot ID: B2IPN5), and *Lactococcus lactis* (Uniprot ID: Q9CHD0) were aligned using ClustalW2^1^ (Larkin et al., 2007). The phylogenetic tree was built using Phylogeny.fr^2^ (Dereeper et al., 2008, 2010).

### Bacterial Strains, Plasmids, and Oligonucleotides

Bacterial strains and plasmids used in this work along with their sources and relevant characteristics are listed in **Supplementary Table S1**. *S. meliloti* strains were routinely grown at 30°C in complex tryptone-yeast TY medium (Beringer, 1974). *E. coli* strains were grown in Luria-Bertani (LB) medium at 37°C. Antibiotics were added to the media when required at the following concentrations (μg/ml): ampicillin (Ap) 200, kanamycin (Km) 50 for *E. coli* and 180 for *S. meliloti*. The sequences of the oligonucleotides used in PCRs or as RNA substrates are provided in **Supplementary Table S2**.

### Overexpression, Purification, and Cross-Linking of Recombinant *Sm*RNase III, E125A, and E125Q Mutants

The *S. meliloti rnc* gene (SMc02652) was amplified by PCR from genomic DNA of strain Sm2B3001 with Phusion high-fidelity DNA polymerase using the primers RNC_F and RNC_R. The purified 741-bp PCR product was double digested with *Nde*I and *Bam*HI and cloned into the histidine tag-containing pET15b vector (Novagen) to yield pET15b-*Sm*RNC. The point mutations E125A and E125Q were introduced into pET15b-*Sm*RNC by site-directed mutagenesis using primers E125A_F, E125A_R, E125Q_F, and E125Q_R originating plasmids pET15b-*Sm*E125A and pET15b-*Sm*E125Q. All constructs were checked by sequencing (STAB Vida, Portugal).

Plasmids were transformed into *E. coli* BL21(DE3) *rec*A*rnc*105 strain to allow the expression of the recombinant proteins (Amarasinghe et al., 2001). This derivative strain of BL21(DE3), carrying an RNase III mutation, was used because it blocks the autoregulation of *Sm*RNase III by the endogenous *E. coli* homolog, resulting in a higher yield of the enzyme upon overexpression (Matsunaga et al., 1996).

Cells were grown at 37°C to an OD_600_ of 0.5 in 150 ml LB medium supplemented with 150 μg/ml ampicillin. At this point, protein expression was induced by addition of 0.5 mM IPTG and bacteria were grown for a further 4 h. Cells were pelleted by centrifugation and stored at -80°C. The culture pellet was resuspended in 3 ml of Buffer A (20 mM Tris-HCl, 500 mM NaCl, 20 mM imidazole, pH 8). Cell suspension was lysed using a French Press at 1,000 psi in the presence of 0.1 mM PMSF. The crude extract was treated with Benzonase (Sigma) to degrade the nucleic acids and clarified by a 30 min centrifugation at 10,000 × *g*. Purification was performed in an ÄKTA FPLC^TM^ system (GE Healthcare). The cleared lysate was subjected to a histidine affinity chromatography in a HisTrap HP column (GE Healthcare) equilibrated in Buffer A. Proteins were eluted by a continuous imidazole gradient up to 500 mM in Buffer A. The fractions containing the purified protein were pooled together, and subjected to gel filtration using a Superdex 200 Increase 10/300 GL (GE Healthcare) column equilibrated with Buffer B (10 mM Tris-HCl, 100 mM NaCl and 1 mM DTT pH 8). The purity of the proteins was verified in a 15% SDS-PAGE gel followed by BlueSafe staining (Nzytech, Portugal). Proteins were quantified using the Bradford method and 50% (v/v) glycerol was added to the final fractions prior storage at -20°C.

To check whether wild-type *Sm*RNase III and its mutant variants dimerize, 1 μg of each of the purified recombinant proteins were incubated in a 10 μl cross-linking reaction with 10 mM HEPES pH 7.4, 250 mM NaCl, 0.1 mM EDTA, 0.1 mM DTT and increasing concentrations (2–20 μg) of DSS (disuccinimidyl suberate). The reaction was performed at room temperature for 30 min and quenched by adding 1 μl of Tris-HCl 1 M pH 7.5 and SDS loading buffer. Samples were boiled during 5 min and then analyzed in a 15% SDS-PAGE gel.

### *In vitro* Production of RNA Substrates

A canonical substrate for RNase III, called R1.1, was generated by *in vitro* transcription of a synthetic DNA template with a commercial promoter oligonucleotide (StabVida), according to a previously described method (Milligan et al., 1987). Briefly, the synthetic DNA template (0.5 μM) and the T7 promoter oligonucleotide (0.6 μM) were annealed in 10 mM of Tris-HCl pH 8.0 by heating for 5 min at 70°C, following by incubation for 30 min, at 37°C. *In vitro* transcription was carried out using “Riboprobe *in vitro* Transcription System” (Promega) and T7 RNA polymerase according to the manufacturer’s instructions and with a molar excess of [^32^P]-α-UTP (3000 Ci/mmol; 10 mCi/ml, which corresponds to ∼3 μM) over non-radioactive UTP (1 μM). In order to remove the DNA template, 1 U of DNase (Promega) was added and incubated 30 min at 37°C.

The mature form of tRNA^Ser^-TGA transcript was also generated. The DNA template for *in vitro* transcription was PCR amplified from chromosomal DNA of *S. meliloti* strain 2011. The phage T7 RNA polymerase promoter sequence was included in the 5′ primer sequence. tRNA^Ser^ was amplified with the primer pair tRNASerT7FW/tRNASerRev.

All the RNA transcripts produced *in vitro* were purified by electrophoresis on a 7 M urea/polyacrylamide gel. The gel slice was crushed and the RNA was eluted overnight at room temperature with elution buffer [3 M ammonium acetate pH 5.2, 1 mM EDTA, 2.5% (v/v) phenol pH 4.3]. The RNA was ethanol precipitated, resuspended in RNase-free water and quantified using the BioPhotometer Plus (Eppendorf). The yield of the labeled substrates (cpm/μl) was determined by scintillation counting.

We also used RNA oligoribonucleotides (StabVida) as substrates in some *in vitro* reactions: 16 and 30 mer alone or both hybridized to the complementary non-labeled 16 mer comp or 30 mer comp to obtain a perfect 16–16 ds duplex, a double-stranded 30–16 ds substrate with a 3′poly(A) tail or the perfect 30–30 ds. The hybridization was performed in a 5:1 (mol:mol) ratio by 10 min of incubation at 80°C followed by 45 min at 37°C. Efficient duplex formation was checked by binding shift assays in each case. Oligoribonucleotides 16 mer and 30 mer were labeled at their 5′ ends with [γ-^32^ATP] and T4 polynucleotide kinase (Ambion) in a standard reaction.

### *In vitro* Activity Assays

The activity assays were performed in a final volume of 50 μl containing the RNase III activity buffer (30 mM Tris-HCl pH 8, 160 mM NaCl, 10 mM MgCl_2_, MnCl_2_, or CaCl_2_, and 0.1 mM DTT) and the RNA substrate (concentrations indicated in the figure legends). As a control, an aliquot was taken prior to the beginning of each assay and was incubated until the end of the assay (without the enzyme). The reactions were started by the addition of the enzyme and further incubated at the indicated temperature. Aliquots of 5 μl were withdrawn at the time-points indicated in the respective figures, and the reactions were stopped by the addition of formamide-containing dye supplemented with 10 mM EDTA. RNase activity assays were carried out at various pH values (pH 5, 6, 7, 8, 9, and 10), and at different Mg^2+^ and Mn^2+^ concentrations (0.1, 1, 5, 10, 50, and 100 mM). Reaction products were resolved in a 7 M urea/polyacrylamide gel. Polyacrylamide concentrations are indicated in the respective figure legends. Signals were visualized by PhosphorImaging and analyzed using ImageQuant software (Molecular Dynamics).

### Construction of the *S. meliloti rnc* Knock-Out Mutant and Phenotypic Tests

RNase III is encoded in the *S. meliloti* chromosome as part of the *lepB-rnc-era* operon. To create an in-frame deletion of the gene, 768-bp *Sac*I/*Bam*HI and 810-bp *Bam*HI/*Pst*I DNA fragments flanking the *rnc* ORF were PCR amplified from Sm2011 (Casse et al., 1979) genomic DNA with primer pairs 1rncKOSacI_F/2rncKOBamHI_R and 3rncKOBamHI_F/4rncKOPstI_R (**Supplementary Table S2**), respectively. Reaction products were restricted with the corresponding enzymes, ligated and inserted as a unique fragment between *Sac*I and *Pst*I sites into the suicide vector pK18*mobsacB* to generate plasmid pKrncKO. This plasmid was mobilized by biparental matting to *S. meliloti* Sm2011 using *E. coli* S17.1 (Simon et al., 1983). Double cross-over events were selected as previously described (Schafer et al., 1994) and the mutants were further checked for the targeted deletion by colony PCR and *Bam*HI restriction of the PCR product. To complement the SmΔ*rnc* mutant, the full-length *rnc* gene was cloned under the control of the IPTG-inducible P*_lac_* promoter as follows. The *rnc* coding sequence was PCR amplified using the pair of primers rncOEI_NdeI_F/BamHI_R. This PCR product was inserted as an *Nde*I/*Bam*HI fragment into the mid-copy plasmid pSRKKm (Khan et al., 2008) to yield pSRKrnc, which was checked by sequencing and conjugated into the SmΔ*rnc* mutant.

For cell morphology inspection, living wild-type and mutant bacteria were placed on 1% TY agarose pads and examined under a Nikon Eclipse Ti-E microscope as described (Robledo et al., 2015). To analyze symbiotic phenotypes, alfalfa (*M. sativa* L. ‘Aragón’) seeds were sterilized with HgCl_2_, washed several times and germinated on 1% agar plates with nitrogen-free mineral solution R&P (Rigaud and Puppo, 1975) for 24–36 h. One cm-long straight seedlings were transferred to glass tubes containing 20 ml R&P hydroponic cultures under axenic conditions. After incubation at room temperature in the dark for 3 days, 24 plants were inoculated with 1 ml of R&P suspension containing 10^6^ bacteria previously grown in complete TY medium (Beringer, 1974) of the wild-type strain Sm2011, the mutant SmΔ*rnc*, or the latter strain harboring either pSRKrnc or the empty vector pSRKKm. Inoculated plants were transferred to a growth chamber programmed for a 16:8 h photoperiod. The number of nodules per plant was recorded every 2–3 days, and 30 days after inoculation plants were harvested and shoot length was measured.

## Results

### The *S. meliloti* Genome Encodes an RNase III Ortholog

**FIGURE 1:**
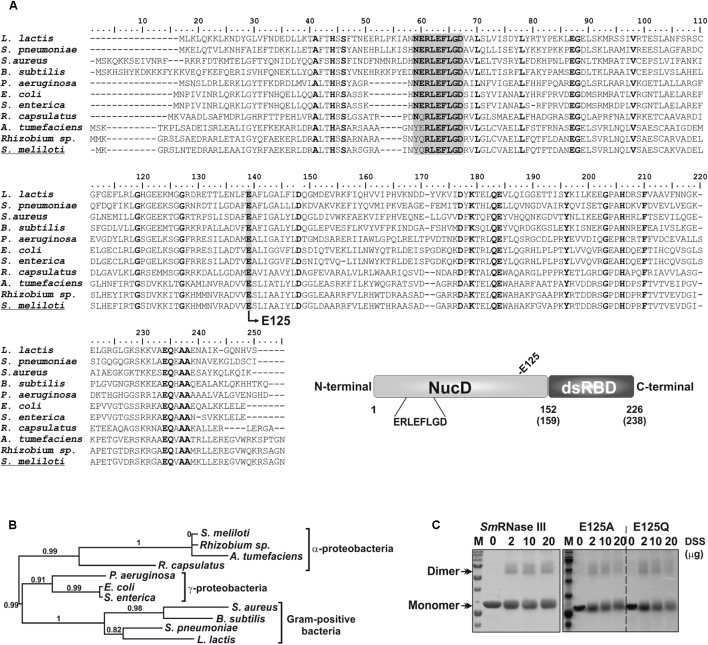
*S. meliloti* RNase III (*Sm*RNase III) protein features. **(A)** Sequence alignment of RNase III from *Lactococcus lactis* (Uniprot ID: Q9CHD0), *Streptococcus pneumonia* (Uniprot ID: B2IPN5), *Staphylococcus aureus* (Uniprot ID: Q2FZ50), *Bacillus subtilis* (Uniprot ID: P51833), *Pseudomonas aeruginosa* (Uniprot ID: Q9XCX9), *Escherichia coli* (Uniprot ID: P0A7Y0), *Salmonella enterica* (Uniprot ID: Q56056), *Rhodobacter capsulatus* (Uniprot ID: Q52698), *Agrobacterium tumefaciens* (Uniprot ID: Q8UGK2), *Rhizobium* sp (Uniprot ID: C3M8S4), and *Sinorhizobium meliloti* (Uniprot ID: M4MQR5). Fully conserved residues are in bold. The signature sequence of these proteins and the mutated E125 residue are shadowed. The schematic representation of the domain organization of RNase III is also shown, indicating the catalytic domain (NucD) (residues 1–152 in *E. coli* protein and 1–159 in *Sm*RNase III) and the dsRNA binding domain (residues 153–226 in *E. coli* protein and 160–238 in *Sm*RNase III). The signature sequence of the catalytic domain is emphasized. **(B)** Phylogenetic tree of RNase III orthologs from the former species. **(C)** DSS-mediated cross-linking of wild-type *Sm*RNase III and its mutant variants E125A and E125Q. Approximately 1 μg of each purified protein were incubated with increasing concentrations of DSS as indicated on top of the panel. “M” corresponds to the protein standard marker. Proteins were visualized by BlueSafe (Nzytech Portugal) staining.

The *S. meliloti rnc* gene (ORF SMc02652 in the genome of the reference strain Rm1021) is predicted to encode an RNase III ortholog, but there are no experimental evidence supporting this prediction. RNase III family members are characterized by conserved structural domains. The bacterial orthologs possess the simplest structures containing an N-terminal catalytic domain (NucD) and a C-terminal dsRNA-binding domain (dsRBD) (Arraiano et al., 2010; Nicholson, 2014). We have aligned the putative *S. meliloti* 238 amino acid-long RNase III polypeptide (*Sm*RNase III) with 10 RNase III orthologs from gram-negative (α-proteobacteria and γ-proteobacteria) and gram-positive microorganisms (**Figure 1A**). This comparison revealed occurrence in *Sm*RNase III of the well-known conserved residues of RNase III family members, including the signature motif ERLEFLGD within the catalytic domain of the enzyme (Arraiano et al., 2010; Nicholson, 2014). This conservation pattern thus supports a *Sm*RNase III domain arrangement characteristic of these endoribonucleases (**Figure 1A**). Nonetheless, phylogenetic clustering of these 11 amino acid sequences grouped *Sm*RNase III with its α-proteobacterial counterparts, in a branch distinguishable from those of the γ-proteobacteria and gram-positive bacteria orthologs (**Figure 1B**).

To further characterize *Sm*RNase III biochemically, we cloned the *rnc* gene and overexpressed the wild-type protein and two mutant variants in the ultraconserved E125 residue in an *E. coli* BL21(DE3) *rec*A*rnc*105 strain (see Materials and Methods). RNase III endoribonucleases typically function as homodimers (Li and Nicholson, 1996). In order to verify if the recombinant *Sm*RNase III undergoes dimerization, the purified protein was incubated with disuccinimidyl suberate (DSS), which reacts with primary amino groups forming stable amide bonds. Fractionation of the cross-linked protein mixtures in a 12% SDS-PAGE gel revealed formation of DSS-dependent species that doubled the size of the *Sm*RNase III monomeric form (∼50 kDa) (**Figure 1C**). Substitution of E125 by either alanine (E125A) or glutamine (E125Q) did not compromise *Sm*RNase III dimerization ability.

### *Sm*RNase III Is a Metal Cofactor-Dependent Endoribonuclease

**FIGURE 2:**
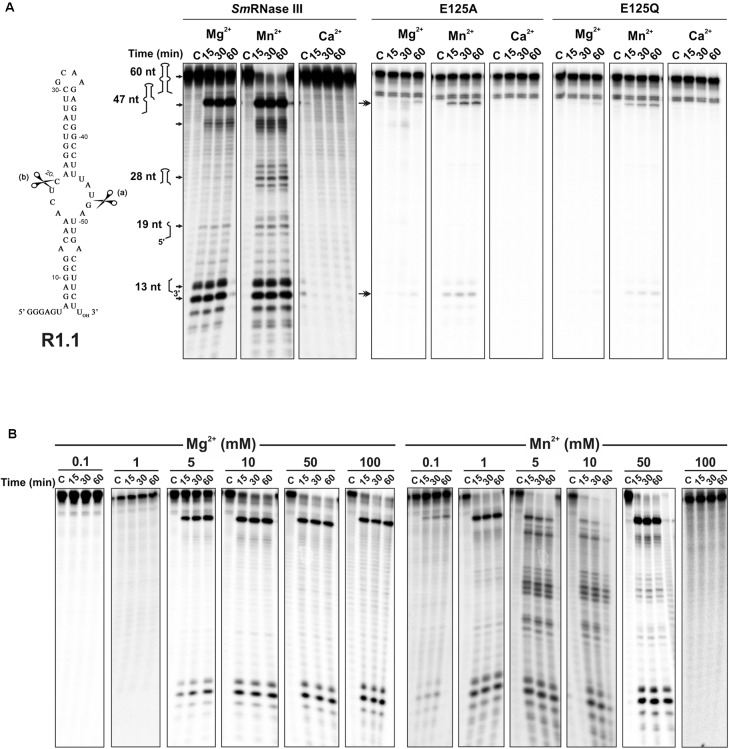
Metal cofactor-dependent activity of *Sm*RNase III on the R1.1 RNA substrate (internally ^32^P labeled). In all cases 500 nM of purified protein were incubated with 0.6 pmol of R1.1 RNA at 37°C in a reaction buffer containing MgCl_2_, MnCl_2_, or CaCl_2_. “C” stands for control reactions without the enzyme. Incubation times are indicated on top of the panels. Reactions were analyzed on 7 M urea/15% polyacrylamide gels. All the experiments were performed at least in triplicate. **(A)** *Sm*RNase III-mediated cleavage of R1.1. Sequence and predicted secondary structure of R1.1 are shown to the left. Major known (*a* and *b*) RNase III cleavage sites on this substrate are indicated by scissors. Panels show R1.1 breakdown patterns upon incubation with the wild-type enzyme or its mutant variants (E125A or E125Q) in the presence of 10 mM concentration of each cofactor. Numbers and schemes on the left of the first series of panels indicate sizes and stretches of the full-length R1.1 transcript and major reaction products (other products are indicated by arrows). Arrows on the left of the second series of panels indicate major cleavage products in reactions with the mutant enzymes. **(B)** Effect of cofactor concentration on *Sm*RNase III activity. The R1.1 substrate was incubated with the wild-type enzyme in the presence of increased concentrations (0.1–100 mM) of MgCl_2_ or MnCl_2_, as indicated on top of the panels.

R1.1 is a structured 60 nucleotides (nt)-long RNA molecule containing an asymmetric (4 nt/5 nt) internal loop, and it comes from the phage T7 early region between genes 1.0 and 1.1. This RNA contains an RNase III primary cleavage site (a) that is recognized *in vivo* and *in vitro*, and a secondary site (b) that is cleaved only *in vitro* (**Figure 2A**) (Dunn and Studier, 1983; Nicholson et al., 1988; Chelladurai et al., 1991, 1993). Therefore, it is commonly used as a model substrate to study RNase III activity *in vitro* for comparison with previous studies.

RNase III is known to cleave R1.1 at these two preferred sites (a and b) in a metal cofactor-dependent manner (Dunn and Studier, 1983; Gross and Dunn, 1987; Li and Nicholson, 1996; Haddad et al., 2013). We first assayed *Sm*RNase III activity on R1.1 in the presence of 10 mM Mg^2+^, Mn^2+^, or Ca^2+^ as divalent metal ions (**Figure 2A**). Both Mg^2+^ and Mn^2+^ supported *Sm*RNase III-mediated catalysis of this substrate, whereas Ca^2+^ did not likely support enzyme activity. Nonetheless, Mg^2+^ and Mn^2+^ distinctly influenced *Sm*RNase III cleavage efficiency and R1.1 breakdown patterns. In Mg^2+^-containing buffer, reactions yielded major R1.1-derived products compatible with cleavage of the substrate at site a (47-nt and 13-nt fragments) similarly to what was observed for RNase III from other microorganisms [data not published and (Sun et al., 2005; Meng and Nicholson, 2008; Haddad et al., 2013)]. Simultaneously, we also observed the presence of other breakdown products (**Figure 2A** and **Supplementary Figure S1**). In this condition, large amounts of the full-length R1.1 molecule were still detectable at the end of the assay (60 min). The same happened when R1.1 was incubated longer (2 h) and with a higher amount of enzyme (1,200 nM) (**Supplementary Figure S2A**). Conversely, Mn^2+^ promoted almost depletion of the substrate and products compatible with an additional cleavage at the canonical b site within R1.1, as it was already reported for other RNase III-like proteins (Meng and Nicholson, 2008; Haddad et al., 2013). The *Sm*RNase III mutant variants E125A and E125Q were largely inactive on R1.1 in the presence of Mg^2+^, but retained partially cleavage ability at site a of the substrate when Mn^2+^ was used as cofactor (**Figure 2A**; right panels). These results indicate that R1.1 cleavage in reactions involving the wild-type enzyme was indeed protein-specific, further suggesting a major role of the ultraconserved E125 amino acid in the metal-dependent catalysis mediated by *Sm*RNase III.

It is known that RNase III-mediated cleavage of R1.1 not only depends on the type of metal cofactor but also on cofactor concentration (Sun and Nicholson, 2001). Therefore, we also tested *Sm*RNase III activity at a wide range of Mg^2+^ and Mn^2+^ concentrations (0.1–100 mM) (**Figure 2B**). Increased Mg^2+^ concentrations favored enzyme activity without altering R1.1 cleavage patterns, with almost total consumption of the substrate at 50 and 100 mM. In contrast, *Sm*RNase III displayed similar efficiency for R1.1 degradation in the window of 1–50 mM Mn^2+^ concentration, whereas higher concentrations of this cofactor (100 mM) rendered the enzyme inactive (**Figure 2B** and **Supplementary Figure S1**). Collectively, these findings confirmed the strictly metal cofactor-dependent activity of *Sm*RNase III on the model R1.1 substrate.

**FIGURE 3:**
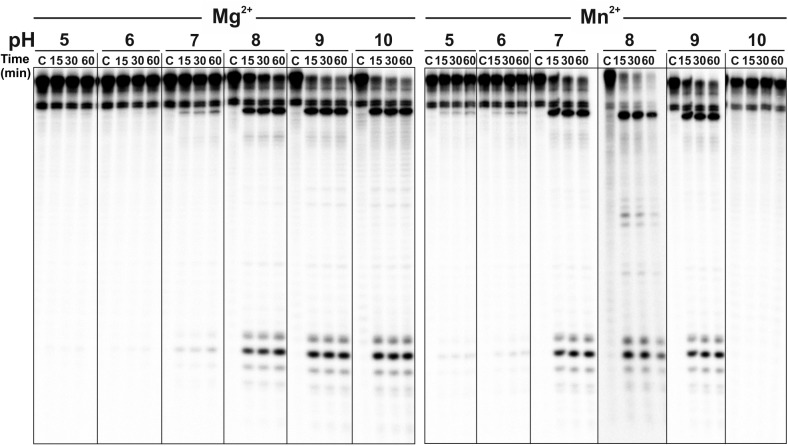
Effect of pH on *Sm*RNase III activity. The wild-type enzyme (500 nM) was incubated with the R1.1 RNA substrate internally ^32^P labeled (0.6 pmol) at 37°C in the presence of 10 mM MgCl_2_ or MnCl_2_ buffered at pH values ranging from 5 to 10 as indicated on top of the panels. “C” corresponds to control reactions without the enzyme. Incubation times are indicated on top of the panels. Reactions were analyzed on 7 M urea/15% polyacrylamide gels. This experiment was performed at least in triplicate.

Another variable that may affect RNase III-mediated catalysis is pH. In a new series of *in vitro* experiments, we assayed *Sm*RNase III for R1.1 cleavage at pH values ranging from 5.0 to 10, in the presence of 10 mM Mg^2+^ and Mn^2+^ (**Figure 3**). Mg^2+^ supported maximal enzyme activity at the highest pH values tested (pH 8.0–10), whereas the use of Mn^2+^ as a cofactor shifted optimal activity to neutral or moderate alkaline conditions (pH 7–9). These data indicate that tolerance of *Sm*RNase III to pH oscillations is also conferred by the metal cofactor.

### *Sm*RNase III Is Double-Strand Specific and Exhibits Different Preference for Endogenous RNA Substrates

**FIGURE 4:**
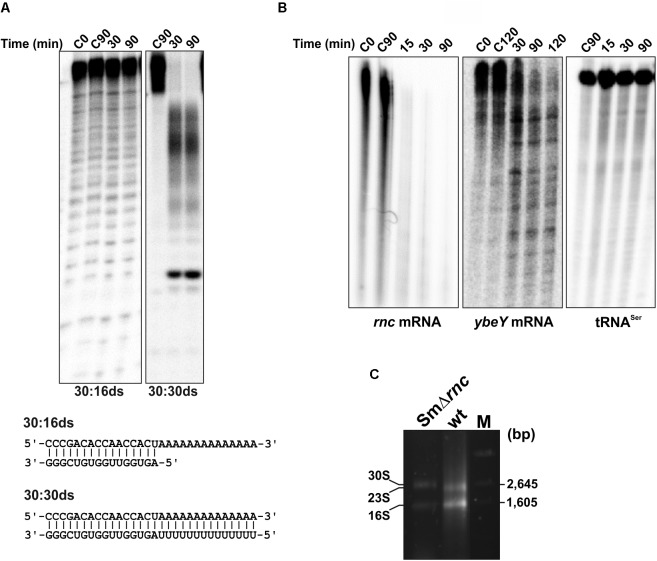
*Sm*RNase III activity on other generic and endogenous RNA substrates. *In vitro* reactions were performed at 37°C at the time-points indicated on top of the panels. “C” corresponds to control reactions without the enzyme. Experiments were performed at least in triplicate. **(A)** Activity on generic dsRNA substrates. The wild-type enzyme (500 mM) was incubated with 0.1 pmol of 16–16ds, 30–16ds or 30–30 ds (5 ′end-labeled) in the presence of 10 mM MgCl_2_. Sequence of each substrate is indicated on bottom of the panels. Reactions were analyzed on 7 M urea/20% polyacrylamide gels. **(B)** Activity on endogenous *S. meliloti* substrates. *Sm*RNase III (500 nM) was incubated with *in vitro* synthetized *rnc* mRNA (0.12 pmol), *ybeY* mRNA (0.15 pmol), or tRNA^Ser^ (0.10 pmol) in the presence of 10 mM MgCl_2_. Reactions were analyzed on 7 M urea/5% (tRNA^Ser^), 8% (*ybeY*), or 10% (*rnc*) polyacrylamide gels. **(C)** Influence of *Sm*RNase III on rRNA maturation. rRNA profiles of the *S. meliloti* wild-type Rm2011 strain and its mutant derivative SmΔ*rnc* analyzed by 0.7% Synergel/0.9% agarose electrophoresis. Sizes of the mature 23S and 16S rRNA species are indicated. 30S denotes the misprocessed 23S rRNA. M, DNA molecular weight marker.

The well-characterized *Ec*RNase III requires a minimal substrate length of ∼11 bps for an efficient, but mostly unspecific cleavage (Nicholson, 2014). To test if *Sm*RNase III behaves similarly with respect to this parameter, we evaluated the ability of the enzyme to degrade a small 16-bp fully dsRNA molecule (16–16 dsRNA) a partially dsRNA with a poly(A) tail (30–16 ds), and a longer 30-bp dsRNA substrate (30–30 ds) in the presence of 10 mM Mg^2+^ (**Figure 4A**). *Sm*RNase III did not react with either the 16–16 ds (data not shown) or the 30–16 ds substrates, whereas the 30–30 ds molecule was fully degraded, with concomitant appearance of likely specific breakdown products (**Figure 4A**). The enzyme neither reacted with the single-stranded versions of these RNAs (i.e., 16 mer and 30 mer oligonucleotides) (**Supplementary Figure S2**). These results confirmed that *Sm*RNase III is a typical double-strand specific endoribonuclease, but with a minimal substrate length requirement different from that of its enterobacterial ortholog.

Bacterial RNase III is known to process or degrade diverse RNA species within the cell, including mRNAs, tRNAs, and rRNAs. Therefore, we also tested *Sm*RNase III activity on a number of *in vitro* transcribed *S. meliloti* native RNA species, i.e., *rnc* and *ybeY* mRNAs and mature tRNA^Ser^ (**Figure 4B**). In the presence of Mg^2+^ as cofactor, the enzyme was very efficient for cleaving its coding mRNA (*rnc*) with rapid consumption of the total substrate. Similarly, *Sm*RNase III degraded the mRNA encoding the endoribonuclease YbeY in the same incubation conditions, but this reaction yielded discrete breakdown products. In contrast, the activity on the mature tRNA^Ser^ transcript was extremely weak. Finally, we compared the rRNA profiles of the wild-type strain and its derivative lacking the endoribonucleases *Sm*RNase III (*Sm*Δ*rnc*) (**Figure 4C**). Accumulation of misprocessed 23S rRNA species (30S rRNA) was evident in mutant bacteria lacking specifically *Sm*RNase III, suggesting that the precursor transcript of the largest rRNA is also a native substrate of this enzyme. Altogether, these findings anticipate a differential contribution of *Sm*RNase III to the turnover and processing of native *S. meliloti* RNA transcripts of diverse biogenesis.

### *Sm*RNase III Influences Free-Living Growth and Symbiotic Performance of *S. meliloti* on Alfalfa Roots

**FIGURE 5:**
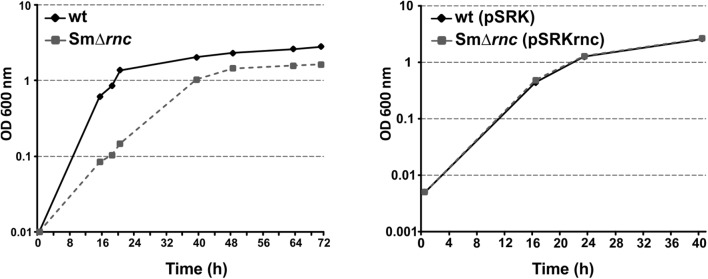
Free-living phenotype of the SmΔ*rnc* mutant. Growth curves of parent (wt; wtpSRK), mutant (SmΔ*rnc*), and complemented (SmΔ*rnc*pSRKrnc) strains as determined by OD_600_ readings of TY cultures. Growth of wild-type bacteria harboring the empty vector pSRK was used as the control reference for the complemented strain.

To further address the biological role of *Sm*RNase III genetically, we characterized the morphology, growth, and symbiotic phenotypes of the *rnc* knock-out mutant, *Sm*Δ*rnc*. Lack of *Sm*RNase III did not alter wild-type length and shape of *S. meliloti* cells, suggesting that this endoribonuclease is not a major determinant of cell division and morphogenesis in this bacterium (data not shown). However, the mutant displayed a markedly delayed growth in complete TY medium with respect to the parent strain, reaching the stationary phase at a lower optical density (**Figure 5**). Growth rate of the mutant was restored to wild-type levels with plasmid pSRKrnc, which expresses the *Sm*RNase III coding gene from an IPTG-inducible P*_lac_* promoter. Of note, this growth phenotype was complemented even in the absence of the inducer, which indicates that a low intracellular accumulation of *Sm*RNase III resulting from uninduced background transcription is enough to fulfill its physiological function.

**FIGURE 6:**
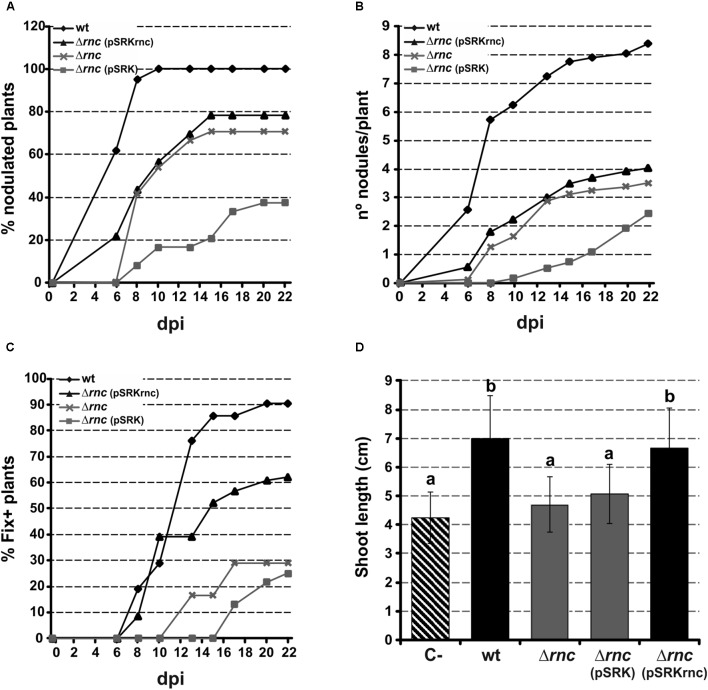
Symbiotic phenotype of the SmΔ*rnc* mutant. Alfalfa plants were inoculated with wild-type (wt), RNase III mutant (Δ*rnc*), complemented (Δ*rnc*pSRKrnc), or control (Δ*rnc*pSRK) strains. The % nodulated plants **(A)**, n° nodules/plant **(B)** and %Fix^+^ plants (i.e., developing at least one pink nodule) **(C)** were recorded at daily intervals and plotted. Shown is a representative example of two independent experiments. Dpi, days post-inoculation. **(D)** Shoot-length of the plants at the end of the assay. Plotted are mean and standard deviation of measurements in a total of 48 plants in two independent experiments. Statistical significance at *P* < 0.05 is indicated by letters. C-, uninoculated plants.

We next investigated the overall impact of *Sm*RNase III in *S. meliloti* symbiotic performance (**Figure 6**). For that, series of 24 alfalfa plants grown hydroponically in test tubes were independently inoculated with the wild-type bacteria, the mutant SmΔ*rnc*, the strain complemented with plasmid pSRKrnc, and a control SmΔ*rnc* derivative carrying the empty vector pSRK. The number of nodules appearing in each plant was recorded at daily intervals to determine nodulation kinetics (% of nodulated plants; **Figure 6A**) and nodulation efficiency (n° nodules per plant; **Figure 6B**) of each strain. This symbiotic test evidenced a marked delay of the *Sm*Δ*rnc* mutant with respect to its parent strain for plant nodulation. At the end of the assay [22 days post inoculation (dpi) of the plants], bacteria lacking *Sm*RNase III hardly nodulated 70% of the inoculated plants, whereas all the plants treated with the wild-type strain developed nodules. Similarly, the average number of nodules induced by the wild-type strain per plant was more than 2-fold than that recorded in *Sm*Δ*rnc*-inoculated plants.

A detailed observation of nodules emerging in roots of wild-type and mutant-inoculated alfalfa plants revealed also a great delay in the appearance of nitrogen-fixing nodules, easily identifiable as pink colored structures because of leghemoglobin expression (**Figure 6C**). At the end of the symbiotic test, more than 90% of plants treated with wild-type bacteria were scored as nitrogen-fixing (i.e., developed at least one pink nodule), whereas this parameter was scarcely 30% in the assay involving the mutant strain. All these symbiotic phenotypes were partially complemented with plasmid pSRKrnc.

The delayed kinetics of appearance of pink nodules suggests that lack of *Sm*RNase III compromises symbiotic nitrogen fixation, which may ultimately affect plant growth. To test this, we collected the alfalfa plants and measured their shoot length (**Figure 6D**). Plants inoculated with the wild-type and complemented strains developed significantly longer shoots than those inoculated with the mutant bacteria, *Sm*Δ*rnc* or *Sm*Δ*rnc* (pSRK), which were similar to those of the control mock-treated plants. Together, these findings suggest that *Sm*RNase III profoundly influences nodulation and the onset of symbiotic nitrogen-fixation in *S. meliloti*.

## Discussion

Ribonucleases are key elements of post-transcriptional regulatory networks that remain poorly characterized in the vast majority of bacterial species. The widespread RNase III family of enzymes are metal cofactor-dependent double-strand specific endoribonucleases, which fulfill prominent roles in bulk mRNA turnover, rRNA/tRNA processing and regulation of gene expression by *trans*-acting and antisense sRNAs. Here, we report the first biochemical characterization of the RNase III ortholog from *S. meliloti* and provide genetic evidence of a major requirement of this enzyme for the establishment of an efficient nitrogen-fixing endosymbiosis between this bacterium and its host legume, alfalfa.

The putative protein product of the *S. meliloti rnc* gene exhibits features common to all RNase III family members, i.e., recognizable catalytic and dsRNA-binding motifs, dimerization potential and strict metal cofactor-dependent catalysis of the generic R1.1 model RNA substrate. Nonetheless, our *in vitro* assays uncovered important differences between *Sm*RNase III and its well-characterized enterobacterial ortholog (*Ec*RNase III) regarding divalent metal ion requirements for optimal catalysis and site-specific R1.1 cleavage. It is known that both Mg^2+^ and Mn^2+^ similarly support efficient *Ec*RNase III-mediated R1.1 cleavage, if provided in the reactions at optimal concentrations, whereas Ca^2+^ does not support catalysis (Sun and Nicholson, 2001). *Ec*RNase III attains optimal activity in the presence of relatively low concentrations of Mg^2+^ (∼2 mM) and Mn^2+^ (0.1–1 mM), but higher Mn^2+^ concentrations (>5 mM) have an inhibitory effect on R1.1 cleavage (Sun and Nicholson, 2001). In contrast, our data showed that *Sm*RNase III demands significantly higher concentrations of both Mg^2+^ (10 mM) and Mn^2+^ (1–50 mM) for R1.1 depletion. As for *Ec*RNase III, increased Mn^2+^ concentrations and the presence of Ca^2+^ blocked the activity of the enzyme. It has been hypothesized that Ca^2+^ does not support *Ec*RNase III catalysis due to its larger radius and different ligand coordination properties (Nicholson, 2014). Unlike *Ec*RNase III, our data indicate that the *S. meliloti* ortholog prefers Mn^2+^ rather than Mg^2+^ as cofactor, which has been already reported for other members of the RNase III family of enzymes (Haddad et al., 2013; Wu et al., 2016). The nature of the metal cofactor seems to be also a major determinant of *Sm*RNase III tolerance to other external variables such as pH. Changes in intracellular pH values are known to affect many biological processes, such as enzymes activity. Thus, we can speculate that modulation of *Sm*RNase III activity at different pH values by metal cofactors, may help bacteria to cope with alkaline stress, namely in the presence of Mg^2+^.

RNase III enzymes have highly conserved residues in the catalytic domain that were suggested to have an important role in activity (Blaszczyk et al., 2004; Akey and Berger, 2005; Ji, 2006; Davies and Walker, 2007). Based on crystallographic data, a two-metal ion catalytic mechanism has been proposed for RNase III. This model suggests that both metals are equally coordinated by four of these residues in the *E. coli* enzyme, i.e., E41 and D45 that belong to the signature box (NERLEFLGDS), and D114 and E117, all within the catalytic motif of the enzyme (Blaszczyk et al., 2004; Sun et al., 2005). The substitution of E117 by either alanine (E117A) or glutamine (E117Q) residues induces minimal structural changes but abolishes the endonucleolytic activity of *Ec*RNase III in the presence of Mg^2+^ without compromising binding to dsRNA (Li and Nicholson, 1996; Dasgupta et al., 1998; Amarasinghe et al., 2001; Haddad et al., 2013). Identical substitutions of the equivalent E117 residue in the *S. meliloti* protein (E125A and E125Q) similarly blocked enzyme activity but supported cleavage at position “*a*” within R1.1 (**Figure 2**) with Mn^2+^ as cofactor. Therefore, it is likely both amino acid substitutions render an environment no longer suitable for Mg^2+^ binding. Crystallographic reports have suggested that Mg^2+^ and Mn^2+^ binding sites have identical coordination in RNase III (Blaszczyk et al., 2001). However, our observations further support the theory of the existence of a third binding site on the enzyme specific for Mn^2+^ (Sun et al., 2005). This interpretation explains the inhibition of *Sm*RNase III activity as Mn^2+^ concentrations increased, whereas Mg^2+^, which is not expected to recognize such Mn^2+^ inhibitory site, is not a limiting factor for catalysis (Sun et al., 2005).

In addition to the primary cleavage site “*a*” within R1.1, which is recognized by RNase III *in vivo* and *in vitro*, it is known that this enzyme cleaves this substrate at a secondary site “*b*” under specific conditions (Gross and Dunn, 1987) (**Figure 2**). Interestingly, we also noticed that *Sm*RNase III is able to cleave R1.1 other sites at regardless the cofactor used, whereas Mn^2+^ specifically promotes cleavage at position “*b*”. These findings anticipate that *S. meliloti* and enterobacterial RNase III orthologs likely recognize different sequence motifs within the RNA substrates. This possibility is further supported by the observation that *Sm*RNase III activity demands longer dsRNA substrates than its enterobacterial counterpart. The *S. meliloti* genome encodes another endoribonuclease called YbeY that exhibits RNase III-like metal cofactor-dependent catalytic features for cleaving structured and dsRNA substrates (Saramago et al., 2017). However, *Sm*YbeY also degrades ssRNA whereas *Sm*RNase III is double-strand specific, which predicts a different contribution of both enzymes to RNA processing and turnover in *S. meliloti*.

*Ec*RNase III is able to regulate its synthesis by a feedback loop that involves cleavage of its own message (Matsunaga et al., 1996). Two lines of experimental evidence support autoregulation of *Sm*RNase III in a similar manner: higher yields of the recombinant protein when it is expressed in a genetic background lacking a functional RNase III (e.g., *E. coli* BL21(DE3) *recArnc*105), and efficient cleavage of its coding mRNA *in vitro*. It is also known that RNase III participates in the maturation of rRNAs (Arraiano et al., 2010). Lack of this enzyme resulted in the accumulation of misprocessed 23S rRNA species (probably 30S rRNA), indicating that *Sm*RNase III is specifically involved in maturation of this housekeeping RNA. RNase III-dependent processing of 23S rRNA has been reported in a number of α-proteobacteria including the *S. meliloti* close relative, *Sinorhizobium fredii*. It likely relies on RNase III-mediated cleavage within intervening sequences occurring specifically in the helix 9 of the α-proteobacterial 23S rRNA precursors (Evguenieva-Hackenberg and Klug, 2000).

The fundamental function of RNases typically influences widely diverse cellular processes. In line with this notion, we have shown that lack of *Sm*RNase III conferred growth and symbiotic defects to *S. meliloti*. Nonetheless, core control of cell division and morphogenesis does not seem to underlie this apparent pleiotropic phenotype, suggesting that *Sm*RNase III participates in specific *S. meliloti* free-living and symbiotic post-transcriptional regulatory networks. Our *in vitro* data are consistent with a proficient activity of *Sm*RNase III under several external conditions, which are known to be relevant for *S. meliloti* lifestyle. Maintenance of metal and pH homeostasis is essential for proper functioning of cells, and has significant implications for microbial adaptations to changing environments. During transition from a free-living state in soil to the intracellular residence within the nodules that *S. meliloti* induce in the roots of its host plant, the essential nutrients Mg^2+^, Mn^2+^, and Ca^2+^ are differentially available and pH fluctuates. The optimum pH for rhizobial growth is neutral or slightly alkaline. Accordingly, acidity severely compromises *S. meliloti* survival in soil, nodule development and symbiotic nitrogen fixation (Ferguson et al., 2013). During early root hair infection, *S. meliloti* is exposed to the reactive oxygen species (ROS) released by the plant host as a defense response (Soto et al., 2009). Manganese has been shown to be important to counteract ROS effects, whereas Mg^2+^ transport is essential for microaerobic nitrogen-fixation in late symbiotic stages (Davies and Walker, 2007; Hood et al., 2015; Hood et al., 2017). It is therefore tempting to speculate on a key role of divalent metal ions in the control of *Sm*RNase III activity *in vivo*. The symbiotic phenotype of bacteria lacking *Sm*RNase III anticipates the involvement of this enzyme in RNA processing and turnover during all steps of the symbiotic interaction. As double-strand specific endoribonuclease, RNase III is expected to mediate gene silencing triggered by sRNAs. The non-coding transcriptome of *S. meliloti* has been characterized in recent years, and a particular bias of antisense transcription to genuine symbiotic genes has been reported (Robledo et al., 2015). Our work, thus provide a base-line for the forthcoming investigation of the discrete participation of *Sm*RNase III in post-transcriptional symbiotic regulatory networks.

## Author Contributions

MS, MR, RGM, JJ-Z, and CMA conceived and designed the experiments, and interpreted the data. JJ-Z and CMA supervised the work. MS, MR, and RGM performed the experiments. MS and JJ-Z wrote the paper. MR, RGM, and CMA critically read the manuscript.

## Conflict of Interest Statement

The authors declare that the research was conducted in the absence of any commercial or financial relationships that could be construed as a potential conflict of interest.

## Footnotes

1 http://www.ebi.ac.uk/Tools/msa/clustalw2/

2 http://www.phylogeny.fr
