# Supplementary material for: Sinorhizobium meliloti RNase III: Catalytic Features and Impact on Symbiosis
Source: Front Genet. 2018 Aug 28;9:350. doi: 10.3389/fgene.2018.00350 (PMC6121014; doi:10.3389/fgene.2018.00350)
Supplement: Supplementary file 1 [file Data_Sheet_1.pdf]

Supplementary Material

Sinorhizobium meliloti RNase III: Catalytic features and impact on Symbiosis

Margarida Saramago, Marta Robledo, Rute G. Matos, José I. Jiménez-Zurdo* and Cecilia M. Arraiano*

*Correspondence:

cecilia@itqb.unl.pt

jijz@eez.csic.es

1.1 Supplementary Figures

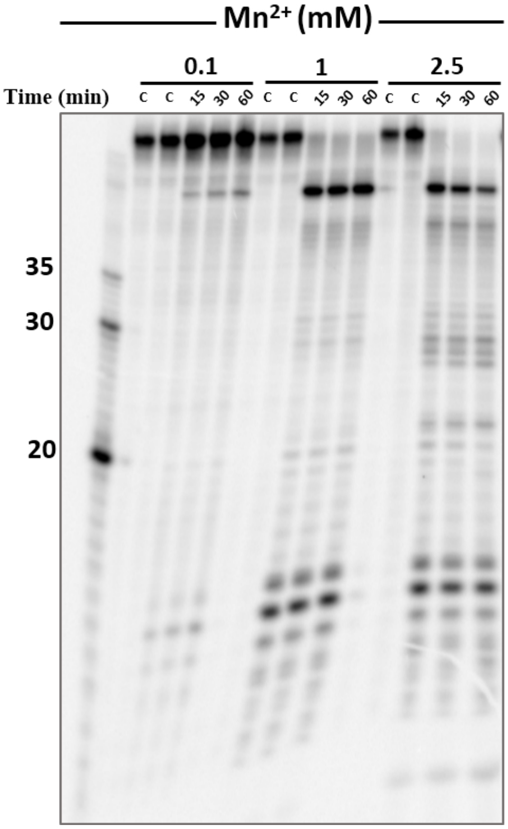

Supplementary Figure S1. 500 nM of purified *Sm*RNase III was incubated with 0.6 pmol of R1.1 RNA at 37°C in a reaction buffer containing 0.1, 1 or 2.5 mM of $MnCl_2$."C" corresponds to control reactions without the enzyme. Incubation times are indicated on top of the panel. Reactions were analyzed on 7 M urea/15% polyacrylamide gels. 5`-end labelled transcripts of known sizes (35, 30 and 20 bases) were included as molecular-weight size marker.

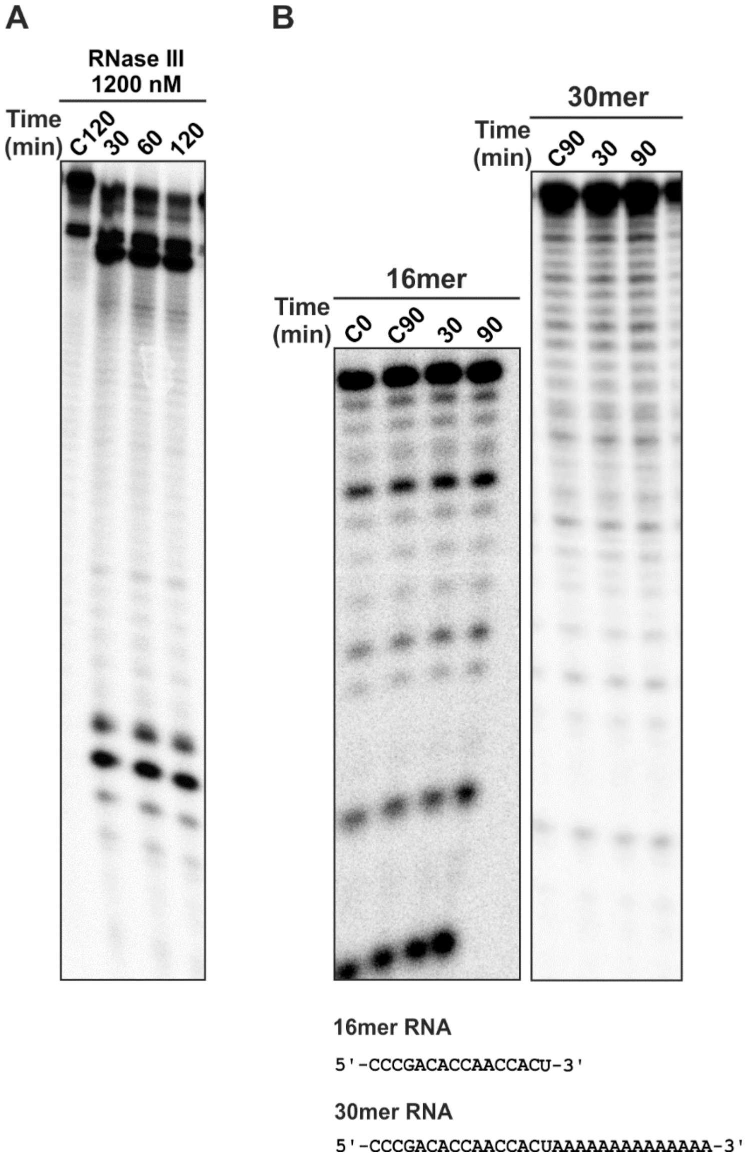

Supplementary Figure S2. (A) *Sm*RNase III (1,200 nM) activity on R1.1 RNA (0.2 pmol). The reaction mix was incubated in the presence of 10 mM $MgCl_2$. "C" corresponds to control reactions without the enzyme. Incubation times are indicated on top of the panels. Reactions were analyzed on 7 M urea/15% polyacrylamide gels. **(B)** Activity of *Sm*RNase III (500 nM) on ssRNA (0.1 pmol of 16mer or 30mer oligonucleotide). Sequence of the RNA substrates are indicated on bottom of the panels. "C" corresponds to control reactions without the enzyme. Incubation times are indicated on top of the panels. Reaction products were analyzed on 7 M urea/20% polyacrylamide gels. This experiment was performed at least in triplicate.

1.2 Supplementary Tables

Table S1. Bacterial strains and plasmids used in this study

Strain/Plasmid	Relevant characteristics	Reference/Source
<u>**Bacteria**</u>		
S. meliloti		
**Sm2011**	Wild-type SU47 derivative derivative; Sm^r	(Casse et al., 1979)
**SmΔ*rnc***	Sm2011 RNase III mutant	This work
E. coli		
**BL21(DE3) *recA**rnc*105**	*rec*A::Tn9Δ*rnc*105; Cm^r	(Amarasinghe et al., 2001)
**DH5α**	F^- *endA1 glnV44 thi-1 recA1 relA1 gyrA96 deoR nupG purB20* φ80d*lac*ZΔM15 Δ(*lacZYA-argF*)U169, hsdR17($r_K^- m_K^+$), λ^-	Invitrogen
**S17-1**	*recA pro hsdR RP4-2-Tc::Mu-Km::Tn7*	(Simon et al., 1983)
<u>**Plasmids**</u>		
**pET15b**	Commercial expression vector; Ap^r	Novagen
**pET15b-*Sm*RNC**	Encodes His-*Sm*RNase III; Ap^r	This work
**pET15b-*Sm*E125A**	Encodes His-*Sm*RNase III_E125A mutant; Ap^r	This work
**pET15b-*Sm*E125Q**	Encodes His-*Sm*RNase III_E125Q mutant; Ap^r	This work
**pK18*mobsacB***	Suicide plasmid in *S. meliloti*, *sacB*, *oriV*, Km^r	(Schafer et al., 1994)
**pKrncKO**	Suicide plasmid for *rnc* deletion; Km^r	This work
**pSRKKm**	Broad-host-range expression vector; Km^r	(Khan et al., 2008)
**pSRKrnc**	pSRKKm derivative encoding *Sm*RNase III; Km^r	This work

Sm^r, streptomycin resistance; Cm^r, chloramphenicol resistance; Ap^r, ampicillin resistance; Km^r, kanamycin resistance

Table S2. Oligonucleotides used in this study

Name	Sequence (5'–3')	Restriction site
tRNASerT7FW	GTTTTTTTTAATACGACTCACTATAGGGACAGGTG GCCGAGTGG	-
tRNASerREV	TGGCGGACAGGGTGGGATTC	-
T7FW	TAATACGACTCACTATA	-
R1.1REV	AAGAAGGTCAATCATAAAGGCCACTCTTGCGAATG ACCTTGAGTTTGTCCCTCTACTCCCTATAGTGAGTC GTATTA	-
30mer	CCCGACACCAACCACUAAAAAAAAAAAAAA	-
30mer comp	UUUUUUUUUUUUUUAGUGGUUGGUGUCGGG	-
16mer	CCCGACACCAACCACU	-
16mer comp	AGUGGUUGGUGUCGGG	-
RNC_F	GTCGTCCACATATGAAGGGCCGC	*Nde*I
RNC_R	GTCATGGGATCCGTCAATTTCCGG	*Bam*HI
E125A_F	GAATGTGCGaGCCGATGTGGTAGcGTCGCTGATC	-
E125A_R	GATCAGCGACgCTACCACATCGGCtCGCACATTC	-
E125Q_F	GAATGTGCGaGCCGATGTGGTAcAGTCGCTGATC	-
E125Q_R	GATCAGCGACTgTACCACATCGGCtCGCACATTC	-
1rncKOSacI_F	GCTAGAGCTCGCAGGATGACAAGGAAGACTG	*Sac*I
2rncKOBamHI_R	AGTCGGATCCTCAGTGGACGACCTTGAACA	*Bam*HI
3rncKOBamHI_F	ATGCGGATCCTTGACGGATATCATGACGGAC	*Bam*HI
4rncKOPstI_R	ATATCTGCAGCGAGATCGCCTTGATCGCCTCG	*Pst*I
rncOEI_NdeI_F	GCCACATATGATGAAGGGCCGCTCGTTGAA	*Nde*I
rncOEI_BamHI_R	TCTCGGATCCCGATTGACCAGTGTCGACTTGC	*Bam*HI

Restriction sites are underlined and base changes with respect to the wild-type sequence are in small letters
